# Hyperelastic Membrane Actuators: Analysis of Toroidal and Helical Multifunctional Configurations

**DOI:** 10.34133/2022/9786864

**Published:** 2022-02-02

**Authors:** Eduardo Perez-Guagnelli, Joanna Jones, Dana D. Damian

**Affiliations:** Department of Automatic Control and Systems Engineering, University of Sheffield, UK

## Abstract

Technologies that provide mechanical assistance are required in the medical field, such as implants that regenerate tissue through elongation and stimulation. One of the challenges is to develop actuators that combine the benefits of high axial extension at low pressures, modularity, multifunction, and load-bearing capabilities into one design while maintaining their shape and softness. Overcoming such a challenge will provide implants with enhanced capacity for mechanical assistance to induce tissue regeneration. We introduce two novel actuators (M2H) built of stacked Hyperelastic Ballooning Membrane Actuators (HBMAs) that can be realized using helical and toroidal configurations. By restraining the HBMA expansion deterministically using a semisoft exoskeleton, the actuators are endowed with axial extension and radial expansion capabilities. These actuators are thus built of modules that can be configured to different therapeutical needs and multifunctionality, to provide anatomically congruent stimulation. We present the design, fabrication, testing, and numerical and experimental validation of the M2H-HBMAs. They can axially extend up to 41% and 32% in their helical and toroidal configurations at input pressures as low as 26 and 24 kPa, respectively. If the axial extension module is used separately, its extension capacity reaches >170%. The M2H-HBMAs can perform independent and simultaneous expansion and extension motions with negligible intraluminal deformation as well as stand at least 1 kg of axial force without collapsing. The M2H-HBMAs overcome the limitations of hyperexpanding machines that show low resistance to load. We envisage M2H-HBMAs as promising tools to perform tissue regeneration procedures.

## 1. Introduction

Soft robots are highly compliant and conformable systems made of materials with similar mechanical properties to those of living tissues [1]. They can perform different motions by combining hyperelastic materials with inextensible substrates or by preprogramming them into their geometries, such as axial extension [2], radial expansion [3], or twisting [4]. These properties provide them with several advantages over conventional rigid robots that have proven to be a compelling alternative to current strategies for the development of medical technologies, such as interacting safely with humans [5]. There has been an increasing interest in the development of medical robots and their components using hybrid soft- and semisoft (a combination of hyperelastic and elastic materials into one system)- [6] based approaches, for either outside of the human body as exosuits [7, 8] or inside, as implants [9]. However, most of these technologies have been focused on wearables [10] that work outside of the body [5] and devices for minimally invasive procedures [11], particularly endoscopic tools [12] and catheters [13]. Robotic implants are devices that can be placed on a target organ or tissue inside of the body to deliver mechanical stimulation based on controlled forces and displacements. The majority of soft robotic implants have focused on treating heart failure [9, 14]; however, these types of devices have the potential to assist in the delivery of a number of therapies.

**Figure 1:**
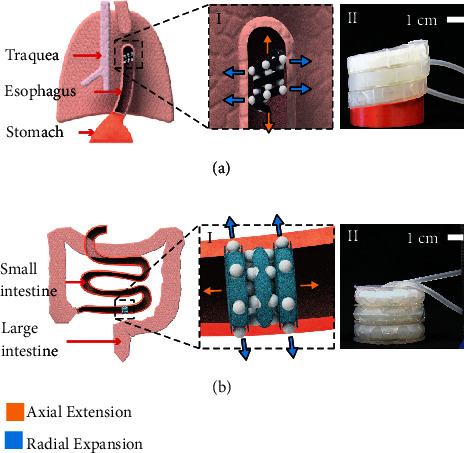
Envisaged application of the Multimodal Hybrid (M2H) and Hyperelastic Ballooning Membrane Actuators (HBMAs). Potential implantation of the M2H-HBMAs inside tubular organs, such as (a) the esophagus and (b) small intestine to treat long-gap conditions. (i) Detailed conceptual views of the helical and toroidal M2H-HBMAs; (ii) the manufactured M2H-HBMAs in a relaxed state. Arrows in the diagrams represent the two motions that the M2H-HBMAs can produce, axial extension and radial expansion.

**Figure 2:**
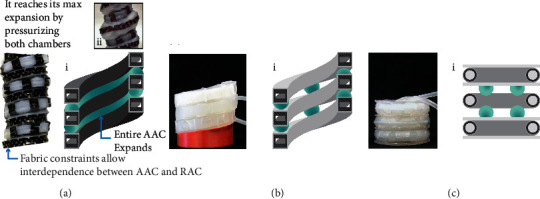
Overview of the design advancements for linear extensibility from (a) our previous work and (b) the HBMA-based helical and (c) toroidal actuator configurations presented in this paper. Inserts (i) show a cross-sectional view of the actuators and their AAC extension principle. Insert (ii) shows a detailed view of this principle in reality.

Regenerative medicine can particularly benefit from the compliance and mechanical support that soft robotic implants can provide. Examples of how this technology can be applied in the field of tissue regeneration include (1) promoting tissue growth of tubular organs of the gastrointestinal (GI) tract caused by congenital defects, such as Long-Gap Esophageal Atresia (LGEA) [15] (Figure 1(a)) or Short Bowel Syndrome (SBS) [16] (Figure 1(b)), and (2) regenerating GI tract tissue after partial resections as a treatment for cancer.

It has been demonstrated that robotic implants that promote tissue regeneration via mechanostimulation in long-term therapies are a promising alternative to traditional approaches in tissue engineering or robot-assisted surgery. Recently, a robotic implant for tissue regeneration in the GI tract was proposed by our group [17]. This implant was capable of growing esophageal tissue up to 77% by exerting a constant force of ~2.5 N over nine days. However, fibrotic response occurred due to the interaction of the rigid parts of the implant with the organ. To decrease fibrotic response, our group developed a flexible version of that implant [18], capable of exerting up to 4 N of force and complying with deflections of up to 3 cm. Nevertheless, despite having flexible components, they are still relatively rigid, potentially not able to decrease fibrosis optimally.

Recent findings demonstrate that fibrosis can be reduced if the surface stiffness of medical implants matches the stiffness of the tissue where they reside [19]. Therefore, we introduced an entirely soft robotic implant for tissue regeneration via mechanotherapy, capable of axially extending 36%, exerting forces up to 7 N, and standing loads of 800 g under pressurization without buckling. Although this design advances existing tissue repair strategies, tissue shortage severity can vary, imposing more drastic tissue elongation and mechanical resistance challenges, which derive into the following requirements:

*Axial extensibility*: to be able to treat different ranges of tissue shortage and therefore reduce the gap between the two stubs or regenerate tissue in a tubular organ affected by, for example, a resection or congenital defect, the design of these new actuators should allow them to highly extend. These actuators should be able to displace from a few to tens of centimeters to support the GI tract. By extending, the actuators will exert traction forces to the host organ and elongate the tissue where it resides (Figure 1). Although the extension of the actuators is only relevant if they are capable of exerting forces to promote cell proliferation, providing them with high axial extensibility can potentially cover a wider range of severities in tissue-shortage conditions. In our previous work [20], we introduced a soft actuator capable of axially extending 36% by pressurizing both of its pneumatic chambers configured in a four coil structure. In this work, we advance those axial extensibility capabilities by achieving higher axial extension while pressurizing only one pneumatic chamber and needing only one coil or level in its configuration (Figure 2).

*Multimodal*: physiological behaviour of human organs involves morphological changes caused by specific anatomical functions, such as peristalsis, performed by the longitudinal and radial muscles in the gastrointestinal tract. To provide anatomically congruent stimulation during mechanical treatment of tubular organs and decrease the risk of pathological responses, the design of these new actuators should provide stimulation on multiple degrees of freedom simultaneously and independently. Given that one envisaged application for the new actuators is to be used inside tubular organs, in addition to being able to axially extend, they should be capable of radial expansion (Figure 1). In this way, the implants could reduce fibrosis by providing massage to the walls of the host organ [21]. Although our previous work [20] introduced an actuator that features multifunctionality, this capability came at the expense of interdependent actuation between its modules, which made accurate control of the device difficult. Here, we advance this feature by providing each of the modules with independent and negligible nonpure motion.

*Modularity*: to increase versatility in their applications across different clinical needs, as well as to adapt to specific anatomical or therapeutical conditions, specialized medical devices should adopt modular strategies. This allows the system to be configured according to the physical requirements of the area where it will reside or the therapy to be performed. In this paper, we advance the concept of modularity through coiling presented in our previous work [20] by introducing a module-stacking approach that allows the assembly of its modules as required.

*Structural strength*: devices that provide mechanical support and stimulation inside the human body, for example, in mechanotherapeutical treatments, need to sustain and exert forces for several weeks [17]. Therefore, they should be capable of resisting considerable loads, while maintaining their structure and softness, preventing the system from collapsing, buckling, damaging its surroundings, or misshaping the target organ. They should also be capable of extending under loaded conditions in order to stimulate the tissue itself. The actuator previously introduced by our group [20] was capable of standing an axial load of 800 g without buckling. In this work, we introduce two new actuators that exceed the load-bearing capacity of our previous design, and we demonstrate their ability to extend under loaded conditions at low pressures.

Although there have been investigations that provide relevant approaches to address one or two of the previously described requirements, the design of soft robotic implants for tissue regeneration via mechanostimulation must comply with all of them. Martinez et al. [2] proposed a paper-elastomer composite pneumatic actuator capable of axially extending 250% and standing up to 1 kg of weight without buckling. Despite its impressive axial extension and load-bearing capabilities, its morphology does not allow for multifunction, a crucial requirement for the physiological well-being of the target organ during mechanotherapy treatment. Digumarti et al. [3] designed a soft pneumatic actuator based on the principle of bellows, which axially extend and radially expand up to 450% and 80%, respectively. However, its radial expansion is only an effect of its axial extension, preventing the device from providing independent axial and radial stimulation, critical in mechanotherapy. Meng et al. [22] designed pneumatic honeycomb-like modular pneumatic structures, capable of axially extending and bending. By adding up to five modules to the structure, the actuator can enhance its extension capabilities. Despite this actuator fulfilling the requirements of axial extensibility, modularity, and structural strength impressively, it could not provide radial stimulation, preventing its use in mechanotherapy. A promising approach was introduced by Blumenschein and Mcngüç [23], in which 3D printed bellow actuators unfold to extend up to 340%. Although this principle has the potential to provide motion in multiple degrees of freedom thanks to its assembly modularity, its working principle does not allow a cylindrical configuration with an empty luminal space, a critical feature for implantation of these types of devices. Lindenroth et al. [24] introduced a pure-extension fluidic actuator that can extend 49% and exert up to 34.83 N/mm of force. Despite its impressive force capabilities, this actuator cannot provide multimodal actuation and is entirely wrapped in a stiff material that may trigger fibrotic response. Cianchetti et al. [25] introduced a manipulator that can axially extend 86.3% and exert 41.4 N of force, but it was not capable of providing independent multimodal actuation.

By encoding the capabilities provided by the compliance of the aforementioned requirements into the design of two novel Multimodal Hybrid (M2H) actuators, we introduce the following contributions to this area of research: (1) introduction of the concept of stacked Hyperelastic Ballooning Membrane Actuators (HBMAs) realized by 3D arrangements of ballooning membranes; (2) a series of numerical analyses to define the M2H-HBMA design features; (3) proposal of two modular and versatile tubular actuator designs, helical and toroidal, based on HBMAs, capable of hyperextensibility and load-bearing; and (4) experimental characterization and validation of the two types of soft actuators.

## 2. Materials and Methods

In this section, we describe the conceptual design, actuation approach, and numerical and experimental analyses we conducted to address the design requirements previously described, as well as to validate and empirically predict the performance of two novel M2H-HBMAs.

### 2.1. Conceptual Design of the M2H-HBMA

**Figure 3:**
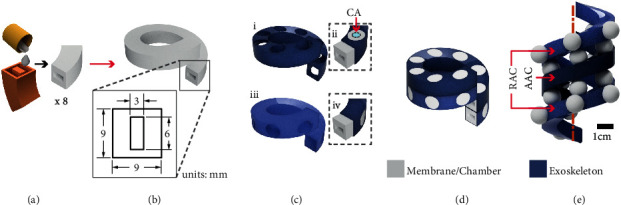
Helical configuration of the stacked balloon concept to shape the helical actuator (HA). (a) Fabrication mold for casting of the units that conform to (b) the helical chambers and a detailed view showing the dimensions of their cross-sectional area. (c) The exoskeletons for (i) AAC and (iii) RAC. Inserts (ii) and (iv) show how we introduced the elastomeric units into the exoskeleton for the AAC and RAC, respectively. (ii) To assemble AAC and RAC, we glued the centre of the AAC membranes to the stacked RAC exoskeleton using chemical bonding (CA). (d) The assembled Helical Hybrid Actuator (HA). (e) Conceptual image of the HA actuated with one turn of the AAC and two turns of the RAC.

**Figure 4:**
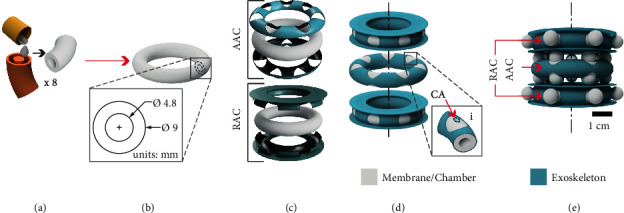
Toroidal configuration of the stacked balloon concept to shape the toroidal actuator (TA). (a) Fabrication mold for casting of the units that conform to (b) the toroidal chambers and a detailed view showing the dimensions of their cross-sectional area. (c) Exploded views of the different parts that conform to the toroidal AAC and RAC. (d) Exploded view showing the assembled independent chambers. Insert (i) shows that to bond the different chambers, we glued the AAC membranes to the RAC's exoskeleton using chemical bonding (CA). (e) Conceptual image of the TA actuated with one AAC and two RACs.

We introduce the concept of stacked Hyperelastic Ballooning Membrane Actuators (HBMAs) [26] in orchestrated 3D expansions, which can be realized using two configurations: helical (Figure 3(e)) and toroidal (Figure 4(e)). Membrane ballooning has been extensively researched in material and mechanical engineering [27–29], mainly in regard to phenomenon modeling and actuation technologies. However, their capabilities in configurable 3D expansions have not been yet addressed. We are using this fundamental membrane ballooning phenomenon with two notable advantages: (1) to compose versatile and load-bearing designs using 3D spatially combined ballooning membranes and (2) to provide those designs with hyperextensibility at reduced initial volume. By restraining the HBMA isotropic expansion deterministically using a semisoft exoskeleton, the helical (HA) and toroidal (TA) actuators are provided with axial extension and radial expansion capabilities. The modules with axial extension capabilities are referred to as Axial Actuation Chambers (AACs). The modules with radial expansion capabilities are referred to as Radial Actuation Chambers (RACs). The exoskeleton can be a flat substrate [26] or 3D structure, but in this work, we will cover the latter. Based on the concept of stacked HBMAs and the previously described requirements, we designed two multimodal actuators (M2H-HBMAs), which conceptual design we describe next. Dimensions and cross-sectional geometries of both M2H-HBMAs are based on our previous work [30] and were selected at a cm scale for ease of fabrication, and because of their simple design, they may be scaled up or down easily in future works. Additionally, we conducted a series of numerical analyses to define the M2H-HBMA design features, such as the comparison between two of the most used unconstrained openings, circular and squared (Section 1), and to evidence the behaviour of the silicone chambers of both actuators without the inclusion of a semisoft exoskeleton (Section 3).

#### 2.1.1. Helical Configuration

The helical actuator (HA) is shaped out of two helical chambers (Figure 3(b)) made of 8 sections (Figure 3(a)) encased into a semisoft exoskeleton (Figure 3(c)) to form the Axial (AAC) and Radial (RAC) Actuation Chambers (Figure 3(e)). The conceptual design of this helical actuator is based on our previous work [20]. In this paper, we advance the fabrication and extension principles by applying an improved HBMA approach (Section 1). When pressurized, the membranes in the AAC expand and shape two balloons, on the top and bottom of the chamber. These balloons displace the stacked levels in the helix, extending it axially. When pressurized, the membranes in the RAC expand and shape balloons in the radial direction (Figure 3(e)).

#### 2.1.2. Toroidal Configuration

The toroidal actuator (TA) is shaped out of toroidal chambers (Figure 4(b)) encased in a two-part semisoft exoskeleton to form the AAC and RAC (Figure 4(c)). Following the concept of stacked balloons, the TA axially extends by expanding balloons out of its AAC, displacing the stacked RACs. Similar to the HA, when pressurized, the membranes in the TA's RAC expand and shape balloons in the radial direction (Figure 3(e)). There are two conceptual differences related to modularity between the HA and TA: (1) In the HA, the height and number of coils depend on the uncoiled length of the two chambers that shape the helix (Figure 3(b)) while in the case of the TA, it will depend on the number of independent units stacked (Figure 4(d)); therefore, one turn in the HA corresponds to one unit in the TA. This equivalence is useful to describe the setup used in Section 3.1.3. (2) Given that the HA relies on only two strands that shape the whole actuator's body, only two air inlets are needed, while in the TA, each unit requires an independent inlet, supplied with air from a common input, independent for the AAC and the RAC. These air input configurations allow the M2H-HBMAs to perform independent and simultaneous axial extension and radial expansion. Diagrams describing the locations of the air inlets are shown in Fig. S8 of Supplementary Materials.

### 2.2. Fabrication Procedure

**Figure 5:**
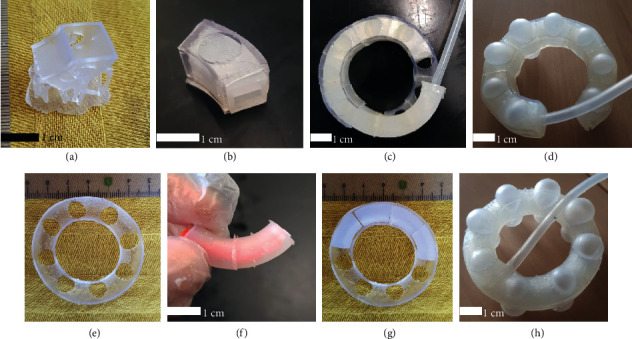
Fabrication procedure. (a) 3D printed HA's exoskeleton section. (b) HA's silicone chamber section inserted into the exoskeleton section. (c) Elastomeric chamber being inserted into the exoskeleton bonded units and (d) an actuated HA's AAC. (e) 3D printed TA's exoskeleton section; (f) toroidal silicone sections being glued using a guiding insert. (g) Demonstration of how the toroidal sections fit into the exoskeleton section before bonding and encasing them to shape an AAC. (h) An actuated TA's AAC.

After defining the conceptual design for the HA and TA based on the design feature numerical analyses (S1 and S2 of Supplementary Materials) and requirements described previously, we proceeded to fabricate them. PLA 3D printed molds (PRUSA, i3 mk3s) were used to cast the chambers and caps of both actuators. Ecoflex 00-30 (Smooth On Inc.) was mixed and defoamed (Thinky ARE-250 Mixer) and then poured into the molds (Figures 3(a) and 4(a)). We cured them at room temperature for four hours. Then, we thermally postcured them at 80°C for two hours and then at 100°C for one hour. After the postcuring process is complete, we bonded the different sections of the helix and torus (Figures 5(b) and 5(f)) until shaping the entire chambers (Figures 3(b) and 4(b)) using uncured silicone. Because adding extra layers of silicone to an elastomeric chamber can create stiffer sections and cause heterogeneous expansion, the bonded surfaces are covered by the exoskeleton, while the ballooning membranes remain unaffected. The exoskeletons were 3D printed out of an elastic resin (Figures 5(a) and 5(e)) with a Shore hardness 50A (Form2, FormLabs©), Young's modulus of 2800 MPa, and Poisson's ratio of 0.43. To assemble the silicone chambers with their respective exoskeletons, we followed different processes, described next.

#### 2.2.1. Assembly of the Helical Actuator

To assemble the parts of the helical actuator, we introduced the silicone chamber into the exoskeleton and pulled gently from one end (Figure 5(c)). To make this procedure easier, we oiled the chamber with vacuum oil, so it reduced the friction between the silicone and the exoskeleton. After the chamber was placed into the exoskeleton, we sealed both ends and added a medical-grade silicone tube as an air inlet that allows pressurization (Figure 5(d)). Finally, to bond the different coils in the helix and to ensure the AAC's balloons push the stacked chambers, we added a drop of cyanoacrylate adhesive between AAC's membranes and RAC's exoskeletons (Figure 3(c), ii).

#### 2.2.2. Assembly of the Toroidal Actuator

To assemble the parts of the TA, we encased a toroidal chamber between the two parts of the AAC and RAC exoskeletons (Figure 5(g)). Then, they were sealed using cyanoacrylate adhesive. Finally, we bonded the AAC (Figure 5(h)) and RAC units in the same manner as described previously for the HA (Figure 4(d), i).

### 2.3. M2H-HBMA Characterization

We conducted a set of bench-top experiments to characterize the extension, multimodality capabilities, and structural strength of the two M2H-HBMAs. In this section, we describe the corresponding setups and protocols.

#### 2.3.1. Control Platform

The control system is composed of a primary printed circuit board that houses the microcontroller, communication and power input, while modular circuit boards contain the pneumatic components for each of the pneumatic chambers: axial (AAC) and radial (RAC). Each of these chambers is inflated and deflated by dedicated DC pumps and closed solenoid valves, respectively. Pressurization of each chamber is triggered by a position proportional integral controller and tracked by Honeywell (ASDXAVX005PGAA5) pressure sensors. Further details about the pneumatic control platform can be found in [31].

#### 2.3.2. Extension Capabilities

**Figure 6:**
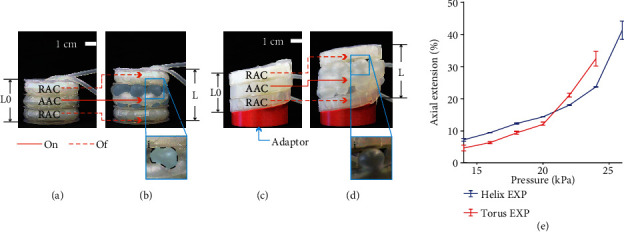
Characterization of extension capabilities of the M2H-HBMAs. (a) The relaxed and (b) pressurized TA at 24 kPa. (c) The relaxed and (d) pressurized HA at 26 kPa. (e) M2H-HBMA extension capability results.

**Figure 7:**
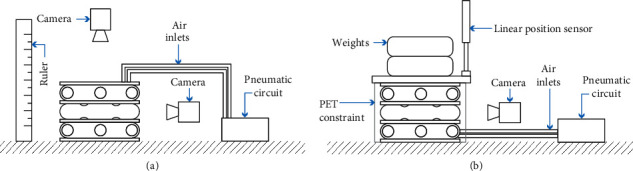
Experimental setups. (a) Axial extension, radial expansion, and intraluminal deformation experiments. The upper camera was only used on the latter. (b) Structural strength experiments. For simplification, only the TA is shown in the diagrams, but the conditions were identical for the HA. Please find a more detailed description of the setup used in this set of experiments in [26].

In this set of experiments, we measured the freeload elongation of the HA and TA when pressurized from 14 kPa with steps of 2 kPa until they failed to find their maximal axial extension. We define the failure point as when the M2H-HBMAs break either at the exoskeleton or membrane level. After reaching each target pressure, it was kept for 4 seconds to ensure the system reached equilibrium. Since its base is not flat, we added an adaptor to the HA to avoid slanted extension (Figure 6(c)). We performed five trials. Extension was recorded and then measured using ImageJ (NIH) (Figure 7(a)).

#### 2.3.3. Pure-Motion Capabilities

The M2H-HBMAs are envisaged to be used as multimodal implantable devices; therefore, their motions should be able to be carried out simultaneously and independently without affecting the physiology of the organ where they reside. For this reason, we conducted two studies. First, we pressurized the RAC of both M2H-HBMAs at 18 kPa. After reaching each target pressure, it was kept for 4 seconds to ensure the system reached equilibrium. Then, we recorded and measured the axial extension provided by the expansion mode of each M2H-HBMAs. Second, since we envisage the use of M2H-HBMAs to internally or externally repair tubular organs, its intraluminal deformation is a relevant feature to test under actuation. Therefore, we activated the extension and expansion modes in both M2H-HBMAs and recorded their behaviour from a top view. Then, we measured the changes in their intraluminal area out of five trials. Both studies were recorded and then analyzed using ImageJ (NIH). A diagram for the entire setup used in the experimental characterization of the actuators is shown in Figure 7(a).

#### 2.3.4. Structural Strength

Finally, since the HA and TA need to exert and sustain forces over a long period of time when used as mechanotherapy implants, their structural strength is an important feature to test. Therefore, to compare the behaviour of both actuators under varying loads, we first conducted a study in which the actuator was initially pressurized and weights were incrementally added. Following on from this, a second study was conducted in which the weights were incrementally added before pressurization, to evaluate the extension of the actuator in active motion under external loads. In the two experiments, the AAC only of either the HA or the TA was used and pressurized to 24 kPa. The AAC chambers were also radially constrained by a sheet of PET, used as a representation of the structural scaffold and generic geometric constraint of the target tubular organ. The displacements of the AACs were measured using the experimental setup from [26] (Figure 7(b)). The experiments were repeated six times.

## 3. Results

### 3.1. M2H-HBMA Characterization

#### 3.1.1. Extension Capabilities

The TA extends up to 32% (13 mm) (Figure 6(b)) with a maximum standard deviation of 2.28% (0.9 mm) before failing at 24 kPa of pressure. The HA extends up to 41% (16 mm) (Figure 6(d)) with a maximum standard deviation of 2.85% (1.08 mm) before failing at 26 kPa of pressure.

If the axial extension module (AAC) was to be used separately, its axial extension capacity increases by >140% and >170% for the TA and HA, respectively (Fig. S9). All percentages of extension presented throughout this section were calculated using the formula (*L* − *L*_0_)/*L*_0_∗100, where *L* is final length and *L*_0_ is the initial length of the actuators, comprising one AAC and two RACs. Figure 6(e) shows a plot of the M2H-HBMA axial extension capabilities at incremental pressures.

#### 3.1.2. Impact of the Actuators' Weight on Balloons

**Figure 8:**
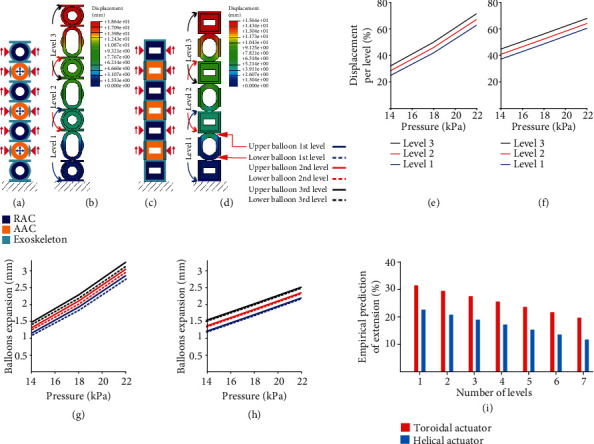
Numerical analysis of the extension capabilities of the M2H-HBMAs. (a, c) Geometry, boundary conditions, load identification, and (b, d) numerical analysis results for the TA and HA, respectively. The axial displacement of the (e) TA's and (f) HA's AACs per level. (g) Plots showing the differences in displacement between upper and lower balloons for every level in the stacked TA and (h) HA. (i) Empirical prediction of the M2H-HBMA axial extension with additional stacked levels.

As can be seen in Figures 6(b) and 6(d), the upper balloons expand more than the lower balloons in both AAC for the HA and TA. This is more evident in the TA. Therefore, with the objective of testing if this corresponds to a physical phenomenon or simply fabrication errors, we proceeded to measure the displacement differences between the upper and lower balloons at different AACs of multilevel M2H-HBMAs using the same settings in a numerical model as for the extension prediction reported in Section 3.1.1. Figures 8(g) and 8(h) verify our observations, also showing that the difference between upper and lower balloons is higher for the TA than for the HA and that these results prevail when stacking more levels.

#### 3.1.3. Numerical Extension Prediction

Figures 8(e) and 8(f) show the stacking effects on axial extension affecting all the levels of three-leveled stacked M2H-HBMAs. The axial extension of the TA's and HA's AACs is decreased by ~4% and ~3.7%, respectively, per added level. By using the results from our experimental (Section 3.1.1) and numerical data for one and multiple stacking levels, we can empirically predict the amount of extension per number of stacked levels, assuming that every level keeps the configuration seen in Figures 8(a) and 8(c), where the two M2H-HBMAs have two RACs and one interlayered AAC. This can be done using the following equation where *E* is the amount of extension for an actuator with *N* levels:

(1) $$\begin{array}{c} E=X-\frac{\left(N-1\right)D}{2}, \end{array}$$

where *X* is the percentage of extension for one level (Figure 6) and *D* is the unit of decrease in extension of the first level in a two-level actuator (Figures 8(e) and 8(f)). This effect is assumed to be constant and is multiplied by the number of upper levels each level has.  Figure 8(i) provides an empirical prediction of the extension for multiple levels of an actuator. This simulated model represents an extreme scenario in which gravitational loading directly affects all levels of the actuator. However, it is worth noting that gravitational effects are dependent on the orientation in which the actuator is used. The silicone chamber models were meshed using quadratic triangular, 2D planar shell elements (CPS6M). To capture the hyperelastic behaviour of silicone, we used the Ogden material model, with the following parameters: *μ*1 = 1.887 × 10^−3^, *μ*2 = 2.225 × 10^−2^, *μ*3 = 3.574 × 10^−3^, *α*1 = −3.848, *α*2 = 0.6632, *α*3 = 4.225, *D*1 = 2.9259, *D*2 = *D*3 = 0 [32]. The exoskeletons were modeled using the mechanical properties provided by the manufacturer, described in Section 2.2.

#### 3.1.4. Pure-Motion Capabilities

**Figure 9:**
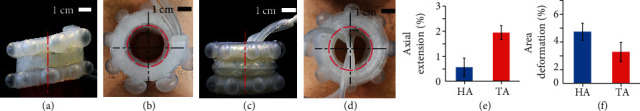
Pure-motion capabilities. (a) The HA and (c) TA, with their RAC pressurized at 18 kPa. (e) Change in extension as a result of expansion for the TA and HA. (b) Top view of the HA and (d) TA pressurized at 24 kPa (AAC) and 18 kPa (RAC). (f) Intraluminal area deformation as a result of expansion and extension for the TA and HA.

The M2H-HBMAs are envisaged to perform specific functions in the body; therefore, their motions should be able to be carried out independently to avoid affecting the therapy and for easier control. By pressurizing the RAC of the HA, the actuator axially extends only 0.5%, while the TA extends by only 1.9%. The intraluminal area of the MHBAs is deformed by 4.8% and 2.3% in the HA and TA, respectively (Figure 9). These results show a negligible nonpure expansion and intraluminal deformation. S3 in Supplementary Materials shows the behaviour of the helical and toroidal chambers without exoskeletons, which evidences the efficiency of the M2H-HBMA exoskeleton designs to allow ballooning membranes (Fig. S9) to expand over 300% without deforming the overall structure.

#### 3.1.5. Structural Strength

**Figure 10:**
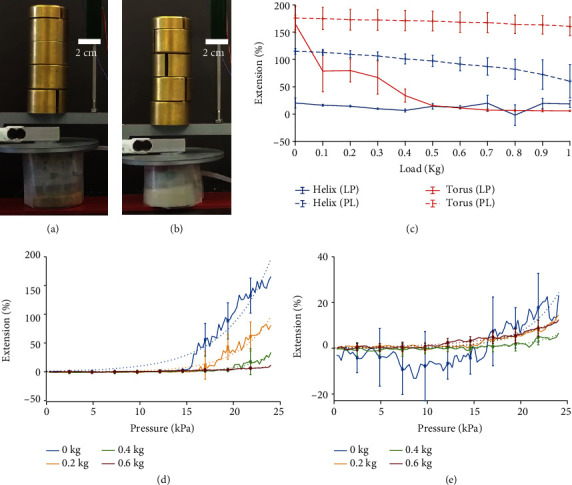
Structural strength test. (a) The TA and (b) HA pressurized at 24 kPa and then loaded by 1 kg. (c) Plot showing the axial extension capabilities of the M2H-HBMAs under varying loads and under two conditions: loaded and then pressurized (LP) and pressurized and then loaded (PL). (d) Extension of the TA and (e) extension of the HA for gradual pressurization to 24 kPa under loads of 0, 0.2, 0.4, and 0.6 kg. The solid lines represent the averaged data with the error bars representing the standard deviation across six trials. The dashed lines represent an exponential curve-fit.

The TA's AAC shows the best performance of the two M2H-HBMAs, with over 150% maximum extension for both the preloaded and postloaded conditions compared to a maximum extension of 115% for the HA's AAC (Figure 10(c)). All percentages of extension presented throughout this section were calculated using the formula (*L* − *L*_0_)/*L*_0_∗100, where *L* is final length and *L*_0_ is initial length of the AAC.

The TA's AAC is also capable of greater extension, when preloaded with extensions of more than 150% as compared to less than 30% for the HA's AAC. The HA's and TA's AACs both perform better when postloaded, showing and maintaining greater extension under heavy loads, demonstrating extensions of 60% and 160% with 1 kg loads, respectively, compared to less than 20% extension for both when preloaded with 1 kg. Finally, the extension of the TA's AAC is considerably more consistent for the postloaded condition showing an average drop of around 15% extension from 0 kg load to 1 kg load compared to around 160% for the preloaded condition.

Figures 10(d) and 10(e) show the extension of the HA's and TA's AAC under gradual pressurization to 24 kPa. Both the HA's and TA's AAC require pressures of around 15 kPa to begin extending under no load conditions. For the TA's AAC, as the load increases, the input pressure needed to begin extension also increases, from around 15 kPa under no load to around 20 kPa for 0.4 and 0.6 kg loads. A similar behaviour is observed for the HA's AAC, although much less distinct due to an overall poorer extension.

Under the preloaded condition, the PET sheet, which represents a structural scaffold of the organ, has little impact on the axial extension, as the loading already restricts the ballooning in all directions. In the postloaded condition, due to the AAC-only inflation, there is limited contact between the balloons and the PET sheet in the pressurizing stage. In the loading phase, the PET sheet prevents the compression of the AAC balloons in the radial direction due to the load and consequently the potential burst of the AAC. While the PET sheet does not model the mechanical properties of a tubular tissue, the sheet plays a generic role in geometrically constraining the actuator.

Further discussions take place in Section 4.

## 4. Discussion

We introduced two novel Multimodal Hybrid Hyperelastic Ballooning Membrane Actuators (M2H-HBMAs), capable of axially extending, radially expanding, and load-bearing resistance with negligible intraluminal deformation and a modular design that can be adapted to different anatomical and therapeutical needs. We envisage the use of these actuators as rehabilitation wearables, when mechanical stimulation is needed outside of the body, or internally, as implants for tissue regeneration. Although various publications address the challenges in the trade-off of force vs. softness and pure-motion vs. multimodality, they do not synthesize the benefits of axial extensibility, multimodality, modularity, and load-bearing capabilities into one design approach, which can be arranged in more than one configuration, as in the TA and HA. In our previous work, we have demonstrated it is possible to provide a device with such capabilities; however, in this work, we advance all of those requirements as described next (Figure 2).

### 4.1. Requirement Compliance

#### 4.1.1. Axial Extensibility

We used numerical analyses to design, analyze, and predict the behaviour of the two M2H-HBMAs, as well as experimental characterization to validate and describe their capabilities. The helical (HA) and toroidal (TA) actuators can axially extend up to 41% and 32%, respectively, by activating only one actuation chamber.

The two Radial Actuation Chambers (RACs) in one level of the M2H-HBMAs are passive modules during axial extension. Therefore, if we derive the obtained axial extension values considering the thickness of the ballooning membranes, we obtain that the membranes showed 250% and 300% of extension in the HA and TA, respectively. If we consider *L*_0_ as the elastomeric chamber, we obtain an extension of 120% and 150% in the HA and TA, respectively (Fig. S9). Because of this, we envisage the development of hyperextensible machines applying the HBMA principle and the M2H actuator stacking module approach.

#### 4.1.2. Modularity

By numerically calculating the decrease in extension per added level and experimentally validating its expansion capabilities, we were able to empirically predict the axial extension for both M2H-HBMAs if we add additional modules (Figure 8). These results validate the compliance to the modularity requirement and demonstrate that the system is still functional and capable of providing high extensibility under multilevel configurations, making it versatile to be adapted to different anatomical or therapeutical needs. We also envisage the use of different modular combinations to provide, for example, pure-expansion only using RACs, or pure-extension using only AACs without the weight of RACs affecting this motion. Additionally, we see the potential to implement bending motions to the TA and HA by selectively pressurizing membranes in the M2H actuators, although this would require adapting the air connections to this application. We recognize that the reconfigurability of the modules is limited by the need to bond them using cyanoacrylate in the current setup, classifying the M2H-HBMAs as assembled modular soft robots [33]. However, we envisage that designers and clinicians will be able to preconfigure the modules to fit their needs before bonding, making use of the M2H-HBMA modularity principles.

#### 4.1.3. Multimodality

The M2H-HBMAs demonstrated exertion of nonpure extension of only <2% and 1% when the RAC is pressurized for the TA and HA and an intraluminal area deformation of <2.5% and 5% when both the RAC and AAC are pressurized for the TA and HA, respectively. These results demonstrate the efficacy of the semisoft exoskeleton in constraining expansion of the silicone chambers, as is highlighted by Fig. S6 in Supplementary Materials and providing multimodal capabilities to the M2H-HBMAs, while keeping softness in their structure.

#### 4.1.4. Structural Strength

The M2H-HBMAs showed the ability to extend when loaded before and after pressurization with up to 1 kg (Figure 10(c)). Both the HA's and the TA's AAC were able to withstand greater loads while maintaining extension levels when loaded after pressurization, showing good structural strength once pressurized. When pressurizing under load, the TA's AAC performed better, showing greater active motion under external loads. The difference in extension when preloaded could be from a more uniform distribution of the load across the balloons of the actuator in the TA's AAC or from the different cross-sectional shape of membranes, with the load only being in contact with the top of the balloon for the circular cross-section in the TA's AAC compared to the whole area for the square cross-section in the HA's AAC.

The extension of the actuator could also be increased through greater pressurization when constrained and under heavier loads, as the level of required input pressure to induce extension was shown to be greater for heavier loads (Figures 10(d) and 10(e)). Given that the presented setup is a geometric-only representation of a tubular organ, further experiments are needed. The effects of a radial constraint for the load-bearing and force exerting capabilities of the TA's and HA's AACs need to be evaluated using tubular constraints that have the mechanical compliance, friction coefficients, and clinical specification according to the medical condition being targeted for treatment. Finally, in the clinical setting, the traction forces exerted on the tissue will also depend on the attachment methods used as an interface between the actuator and the organ [34].

### 4.2. Future Directions

#### 4.2.1. Resilience

The type of forces required to promote tissue growth is still unknown. However, as mentioned in Section 1, Damian et al. [17] achieved the elongation of esophageal tissue in vivo, providing relevant quantitative data, which we can use to assess the applicability of the M2H-HBMAs, such as the actuators having operated ~9 days inside the body to elongate 77% while exerting a constant force of ~2.5 N. Recurring actuation during those 9 days may lead to weakening or bursting of the soft matrix internal walls, causing failure. We acknowledge that, although the robots presented in this work could provide real-time information about their extension, force, and pressure conditions in bench-top tests, their use inside the body would be affected by the aforementioned disadvantage. Nevertheless, different approaches have been developed by the soft robotics community to overcome this challenge and make soft robots resilient, such as self-healing techniques [35] and advanced proprioceptive sensing strategies [36] that could be adopted in future works.

#### 4.2.2. Biocompatibility

The materials used to fabricate the M2H-HBMAs were selected due to their inherent compliance, stretching capacity, ease of use, low cost, high availability, and versatility that allow rapid prototyping; however, they are not biocompatible, which prevents its use in direct contact with organs and tissues. There are two approaches to overcome this limitation and expand the reach of this work outside of proof-of-concept: (1) encapsulation and (2) replacement of the elastomeric matrix using a biocompatible material, such as silastic [17].

#### 4.2.3. Fabrication and Modeling

Although the manufacturing process steps are straight-forward and well defined, they require performing manual procedures that might compromise the reliability of the system, such as bonding the AAC membrane to the RAC surfaces (Figures 3(c), ii and 4(d), i) or assembling the exoskeletons (Figure 5(a)). Using multimaterial additive manufacturing technologies to print simultaneously, chambers and exoskeletons can overcome this challenge. Additionally, further numerical analysis that considers a more realistic 3D contact interaction among modules and ballooning behaviour under load is needed.

#### 4.2.4. Exoskeleton Stiffness/Extension Dependency

The modular approach used in the TA is not affected by the mechanical properties of its exoskeleton as long as it has a higher elastic modulus than its silicone chamber and membranes. However, the stiffness of the HA's exoskeleton and its extension capabilities are interdependent. Decreasing the stiffness of the HA exoskeleton, for example, by varying its wall thickness or material's shore hardness, might achieve higher or lower axial extension rates. This is caused by the resistance of the helix to increase or decrease its pitch. An analytical model that expresses the relationship between the HA's pitch behaviour and axial extension as a function of its exoskeleton stiffness can overcome this challenge.

#### 4.2.5. Implant-to-Tissue Force Evaluation

While the M2H-HBMA's axial extension is promising and although we have demonstrated that the M2H-HBMAs can sustain at least 1 kg of force while axially extending, this should be evaluated as a function of the forces that they can apply to biological tissue in order to confirm that their design is capable of addressing the mechanotherapy requirements. Furthermore, in order to fully demonstrate the suitability of M2H-HBMA's design as a tissue repair tool, we need to evaluate their reliability over several weeks [17].

In conclusion, we have designed and conducted a series of numerical analyses to define the M2H-HBMA design features and experimentally validated the design of two M2H-HBMAs that can sustain considerable loads and provide pure-extension motions with minimal intraluminal deformations. These actuators achieve axial extension by using a hyperelastic ballooning membrane principle in combination with a stacked balloon approach in helical and toroidal configurations. We envisage the use of these tools to assist tissue repair or function recovery in surgical, implantable, and wearable applications by providing mechanical stimulation to the limb, organ, or tissue where they are placed. Future work includes (1) a systematic study of the effect of external forces on the actuator, (2) ex vivo testing to analyze tissue and organ response to mechanical stimulation provided by the M2H-HBMAs, and (3) the development of an analytical model to describe their behaviour.

## Supplementary Materials

Supplementary Materials S1: effect of the exoskeleton's unconstrained area geometry on expansion and stress in the membrane. S2: contact analysis. S3: intraluminal deformation. S4: comparison to braided pure-extension actuators to provide a preliminary comparison between the actuators presented in this work in terms of extension and strength. Further details on the design and operating principle of the BAs can be found in [37].

## References

[1] Rus D., Tolley M. T. (2015). Design, fabrication and control of soft robots. *Nature*.

[2] Martinez R. V., Fish C. R., Chen X., Whitesides G. M. (2012). Elastomeric origami: programmable paper-elastomer composites as pneumatic actuators. *Advanced Functional Materials*.

[3] Digumarti K. M., Conn A. T., Rossiter J. (2017). Euglenoid-inspired giant shape change for highly deformable soft robots. *IEEE Robotics and Automation Letters*.

[4] Yan J., Zhang X., Xu B., Zhao J. (2018). A new spiral-type inflatable pure torsional soft actuator. *Soft Robotics*.

[5] Payne C. J., Wamala I., Abah C. (2017). An implantable extracardiac soft robotic device for the failing heart: mechanical coupling and synchronization. *Soft Robotics*.

[6] Zhang J., Wang T., Wang J. (2020). Geometric confined pneumatic soft–rigid hybrid actuators. *Soft Robotics*.

[7] Asbeck A. T., de Rossi S. M. M., Holt K. G., Walsh C. J. (2015). A biologically inspired soft exosuit for walking assistance. *The International Journal of Robotics Research*.

[8] Samper-Escudero J. L., Contreras-González A. F., Ferre M., Sánchez-Urán M. A., Pont-Esteban D. (2020). Efficient multiaxial shoulder-motion tracking based on flexible resistive sensors applied to exosuits. *Soft Robotics*.

[9] Roche E. T., Horvath M. A., Wamala I. (2017). Soft robotic sleeve supports heart function. *Science Translational Medicine*.

[10] Bützer T., Lambercy O., Arata J., Gassert R. (2020). Fully wearable actuated soft exoskeleton for grasping assistance in everyday activities. *Soft Robotics*.

[11] Runciman M., Darzi A., Mylonas G. P. (2019). Soft robotics in minimally invasive surgery. *Soft Robotics*.

[12] Son D., Gilbert H., Sitti M. (2020). Magnetically actuated soft capsule endoscope for fine-needle biopsy. *Soft Robotics*.

[13] Kumar N., Wirekoh J., Saba S., Riviere C. N., Park Y. L. (2020). Soft miniaturized actuation and sensing units for dynamic force control of cardiac ablation catheters. *Soft Robotics*.

[14] Saeed M. Y., van Story D., Payne C. J. (2020). Dynamic augmentation of left ventricle and mitral valve function with an implantable soft robotic device. *JACC: Basic to Translational Science*.

[15] Foker J. E., Kendall Krosch T. C., Catton K., Munro F., Khan K. M. (2009). Long-gap esophageal atresia treated by growth induction: the biological potential and early follow-up results. *Seminars in Pediatric Surgery*.

[16] Spencer A. U., Kovacevich D., McKinney-Barnett M. (2008). Pediatric short-bowel syndrome: the cost of comprehensive care. *The American Journal of Clinical Nutrition*.

[17] Damian D. D., Price K., Arabagi S. (2018). In vivo tissue regeneration with robotic implants. *Science robotics*.

[18] Atwya M., Kavak C., Alisse E., Liu Y., Damian D. D. (2020). Flexible and expandable robot for tissue therapies-modeling and design. *IEEE Transactions on Biomedical Engineering*.

[19] Carnicer-Lombarte A., Barone D. G., Dimov I. B. (2019). Mechanical matching of implant to host minimises foreign body reaction. *bioRxiv*.

[20] Perez-Guagnelli E., Jones J., Tokel A. H. (2020). Characterization, simulation and control of a soft helical pneumatic implantable robot for tissue regeneration. *IEEE Transactions on Medical Robotics and Bionics*.

[21] Huang C., Holfeld J., Schaden W., Orgill D., Ogawa R. (2013). Mechanotherapy: revisiting physical therapy and recruiting mechanobiology for a new era in medicine. *Trends in Molecular Medicine*.

[22] Meng C., Xu W., Li H., Zhang H., Xu D. (2017). A new design of cellular soft continuum manipulator based on beehive-inspired modular structure. *International Journal of Advanced Robotic Systems*.

[23] Blumenschein L. H., Mcngüç Y. Generalized delta mechanisms from soft actuators.

[24] Lindenroth L., Housden R. J., Wang S., Back J., Rhode K., Liu H. (2019). Design and integration of a parallel, soft robotic end-effector for extracorporeal ultrasound. *IEEE Transactions on Biomedical Engineering*.

[25] Cianchetti M., Ranzani T., Gerboni G., De Falco I., Laschi C., Menciassi A. Stiff-flop surgical manipulator: mechanical design and experimental characterization of the single module.

[26] Herzig N., Jones J., Perez-Guagnelli E., Damian D. D. Model and validation of a highly extensible and tough actuator based on a ballooning membrane.

[27] Treloar L. (1944). Strains in an inflated rubber sheet, and the mechanism of bursting. *Rubber Chemistry and Technology*.

[28] Feng W. W., Pangnan H. (1975). On the general contact problem of an inflated nonlinear plane membrane. *International Journal of Solids and Structures*.

[29] Baumgartner R., Kogler A., Stadlbauer J. M. (2020). A lesson from plants: high-speed soft robotic actuators. *Advanced Science*.

[30] Perez-Guagnelli E., Damian D. D. (2021). Deflected Versus Preshaped soft pneumatic actuators: a design and performance analysis Toward reliable soft robots. *Soft Robotics (Conditional Acceptance)*.

[31] Perez-Guagnelli E. R., Nejus S., Yu J., Miyashita S., Liu Y. Q., Damian D. D. Axially and radially expandable modular helical soft actuator for robotic implantables.

[32] Agarwal G., Besuchet N., Audergon B., Paik J. (2016). Stretchable materials for robust soft actuators towards assistive wearable devices. *Scientific Reports*.

[33] Zhang C., Zhu P., Lin Y., Jiao Z., Zou J. (2020). Modular soft robotics: modular units, connection mechanisms, and applications. *Advanced Intelligent Systems*.

[34] Perez-Guagnelli E., Jones J., Damian D. D. Evaluation of a soft helical actuator performance with hard and soft attachments for tissue regeneration.

[35] Roels E., Terryn S., Brancart J., Verhelle R., van Assche G., Vanderborght B. (2020). Additive manufacturing for self-healing soft robots. *Soft Robotics*.

[36] Helps T., Rossiter J. (2018). Proprioceptive flexible fluidic actuators using conductive working fluids. *Soft Robotics*.

[37] Connolly F., Polygerinos P., Walsh C. J., Bertoldi K. (2015). Mechanical programming of soft actuators by varying fiber angle. *Soft Robotics*.

