# Supplementary material for: Hyperelastic Membrane Actuators: Analysis of Toroidal and Helical Multifunctional Configurations
Source: Cyborg Bionic Syst. 2022 Feb 2;2022:9786864. doi: 10.34133/2022/9786864 (PMC9494722; doi:10.34133/2022/9786864)
Supplement: Supplementary Materials — S1: effect of the exoskeleton's unconstrained area geometry on expansion and stress in the membrane. S2: contact analysis. S3: intraluminal deformation. S4: comparison to braided pure-extension actuators to provide a preliminary comparison between the actuators presented in this work in terms of extension and strength. Further details on the design and operating principle of the BAs can be found in [37]. [file 9786864.f1.zip › M2H_HBMAs__Supporting Information_Clean Version.pdf]

Supplementary Materials

S1 Effect of the exoskeleton's unconstrained areas geometry on expansion and stress in the membrane

To maximize the expansion rates of the membranes that consequently benefits the overall motion of the M2H-HBMAs, we developed a numerical model that analyzes the effect of the exoskeleton's unconstrained area geometry on expansion and stress in the membrane. These are the exposed areas where the membrane will expand out of the exoskeleton (Fig. S1(a-b)). Given that the interaction between the exoskeleton and the silicone balloon impacts on the behaviour of the latter, this is a relevant design parameter to consider. For example, higher stress concentrations in the exoskeleton-membrane interface might lead to bursting of the balloon. In this analysis, we considered circular (Fig. S1(a,i)(b,i)) and squared geometries (Fig. S1(a,ii)(b,ii)) because they are the most basic shapes for the unconstrained areas. Also, circular and squared openings were selected because they produce the least amount of stress in the elastomeric matrix and for consistency with our previous works respectively. For the shape of the chamber of the fluidic actuator contained in the exoskeleton, we also selected circular and squared geometries, two of the most common shapes for fluidic actuators. The used dimensions (Fig. S1(e-f)) are set for a prototype that at this point would fit to be mounted externally to human tubular organs, though scaling down is envisaged as a subsequent phase of development. Fig. S1(c,d) shows the boundary and load conditions, as well as Δ, used to measure performance efficiency with the following equation:

$$i = \frac{\Delta}{\sigma} \tag{2}$$

where Δ is the maximum expansion of the ballooning membrane when pressurized and σ is the highest stress concentration value in the interface between the ballooning membrane and the constraining surface. The most efficient combination of chamber and constraint geometry will allow the silicone to expand higher with lower amount of stress, potentially increasing the actuator's fatigue life. The technical implication is that the more the membrane expands without bursting, the higher the axial extension and radial expansion of the actuator. An inefficient configuration is represented by either, high expansion rates with high stress concentrations, low expansion rates with low stress, or low expansion with high stress concentrations. The settings for the simulation are described in Section S3. The material modeled for the constraining surface is a 3D printed polymer, further described in Section 2.2.

As can be seen in Fig. S2(a), both exoskeleton's unconstrained sections geometries in the simplification of the squared chamber, showed to achieve the highest expansion among all the designs when they are pressurized. However, also both of the unconstrained sections in the simplification of the circular chambers, showed the lowest stress concentrations around the interface between the exoskeleton and the ballooning membrane (Fig. S2(b)) under the same pressure conditions. By using Eq. 2, we identified the design with higher efficiency in terms of expansion and stress. Fig. S2(c) shows that the circular unconstrained geometries are the best trade-off between expansion and stress

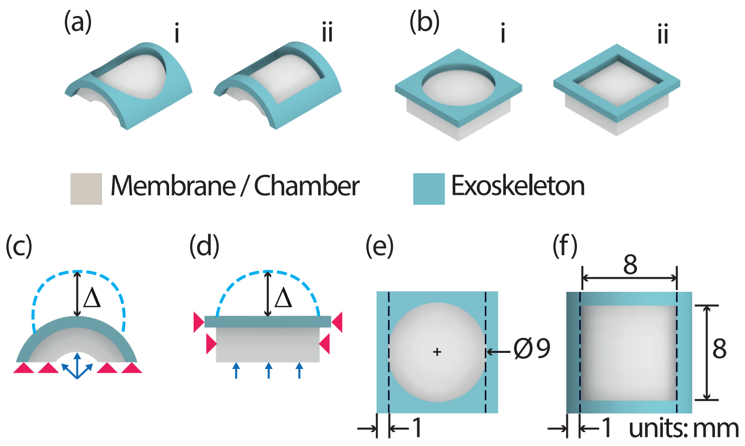

Figure S1: Setup for the numerical analysis of the unconstrained area's geometry in the exoskeleton to assess the membrane expansion and stress concentrations. The simplified geometries tested in this analysis for the (a) circular and (b) squared chambers. For both shapes, the inserts i and ii show circular and squared unconstrained areas respectively. Identification of the load, boundary conditions and measured displacement (Δ) used in this analysis for the (c) circular and (d) squared chambers. General dimensions of the tested geometries for the (e) circular and (f) squared chambers.

concentrations for both M2H-HBMAs. This means that the design of both actuators should include circular unconstrained areas for the membrane to expand instead of squared to reduce the risk of tearing without compromising expansion rates.

S2 Contact Analysis

Expansion of the membranes enables the motion of the M2H-HBMAs and their envisaged configuration is a set of stacked modules. Due to this, it is relevant to describe the interaction between the ballooning membrane and the surfaces they are pushing to achieve axial extension or to provide stimulation through radial expansion. We developed a numerical model to determine the most efficient interface between the ballooning membrane and the exoskeleton of the stacked chambers in function of their contact pressure and exerted force.

For this analysis, we used two types of interface shapes: a convex shape (Fig. S3(a)) and a flat shape (Fig. S3(b)). We modeled a simplified version of the membrane and exoskeleton and placed them below the convex and flat shapes with three variations for each of them: 1 ((Fig. S3(c,f)), 2 (Fig. S3(d,g)) and 3 mm ((Fig. S3(e,h)) of separation between the relaxed membrane and the contact surface. These incremental levels of separation allow us to define the evolution in contact pressure and force in stages. The settings for the simulation are described in Section S3. The material properties for the constraining shapes are described in Section 2.2. The boundary conditions and load identification are shown in Fig. S3.

The convex shape (Fig. S4(a-c)) produces up to $\sim$ 30 kPa of contact pressure, while the flat shape produces only $\sim$ 17 kPa (Fig. S4(d-f)), almost 50% lower contact pressures. Although these results do not necessarily imply that the convex shapes are $\sim$ 50% more prone to failure than the flat shapes

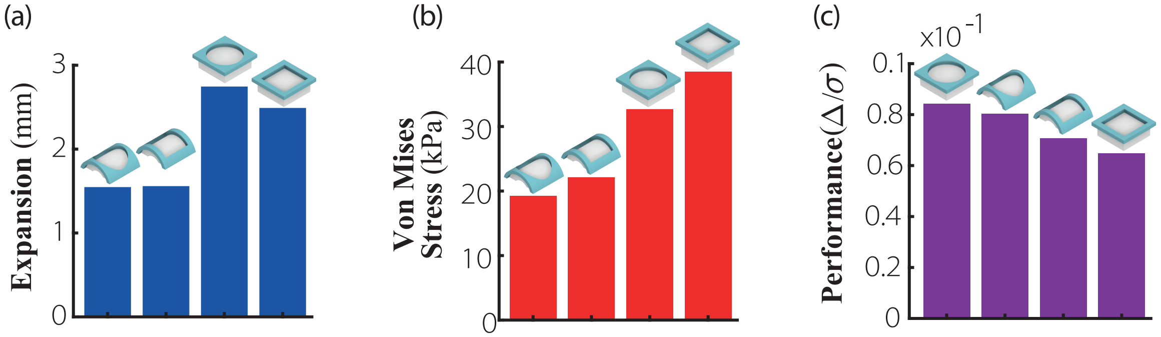

Figure S2: Effect of the exoskeleton's unconstrained areas geometry on (a) expansion (Δ) and (b) stress (σ) of ballooning membranes when pressurized at 32 kPa. (c) Performance index of the expandable ballooning membrane, showing that circular unconstrained areas yield higher expansion rates at lower stresses in comparison to the squared unconstrained areas.

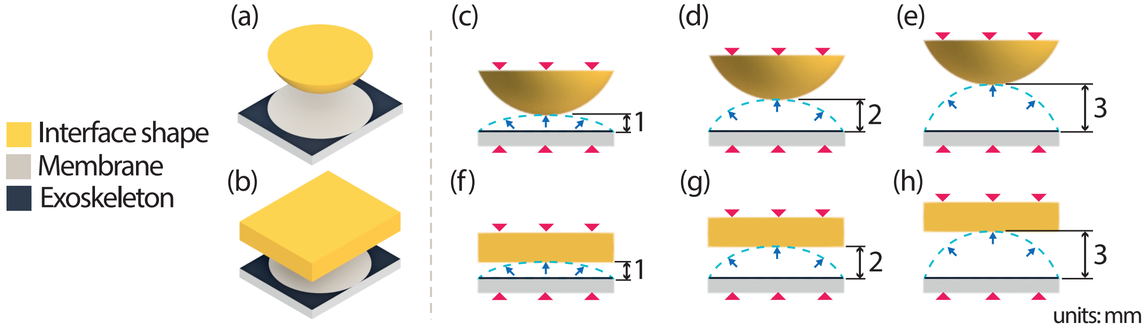

Figure S3: Numerical modeling setup for the contact analysis of a pressurized membrane. Isometric view of the geometries and components used in the analysis for the (a) convex and (b) flat interfaces. Diagrams showing the location of the boundary conditions and forces for 3 scenarios, varying the distance from 1 to 3 mm between a silicone membrane and (c-e) convex and (f-h) flat interfaces.

as a result of contact interaction, lower stresses in the silicone membrane should contribute to increase longevity of the system. Despite exerting higher contact pressures, the ballooning membrane exerts higher forces, of up to 30% against the flat shape than the convex one (Fig. S4 g). This is relevant for the design of the two M2H-HBMAs, as their axial extension relies on ballooning membranes that push upwards downwards the stacked levels above and bellow the Axial Actuation Chambers (AACs). Additionally, Fig. S4(a-c, ii) shows a higher deformation of the membrane for the convex interface than for the flat interface Fig. S4(d-f, ii). These results motivated the inclusion of the flat surface on top and bottom of the TA's RAC (Fig. 4(d)). In this way, the AAC's ballooning membranes will be pushing the stacked RACs using a flat interface.

S3 Intraluminal Deformation

Given that the M2H-HBMAs could be implanted intraluminally or extraluminally, it is highly relevant to analyze their luminal behavior under pressurization. To demonstrate the efficacy in the

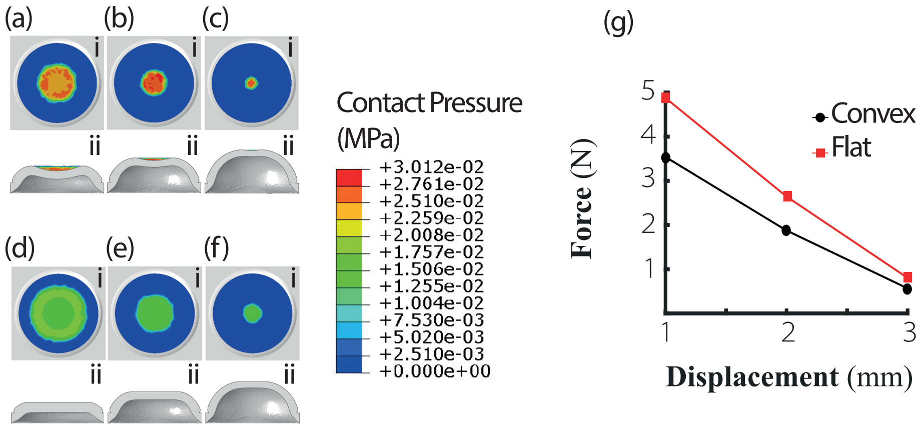

Figure S4: Contact analysis results. Top undeformed-view of the plotted contours showing contact pressure concentrations in the ballooning membrane against (a-c) the convex shape (d-f) and the flat shape, with a separation of 1, 2 and 3 mm respectively. Inserts ii of (a-c) show a deformed side cross-sectional view of the convex shape. Inserts ii of (d-f) show a deformed side cross-sectional view of the flat shape. (g) Comparison of the evolution in the contact force exerted for both contact interfaces.

design of the TA and HA exoskeletons to keep their intraluminal geometry, we numerically modeled their silicone chambers (Fig. 3(b) and (Fig. 4(b)), unconstrained and under pressurization. Typically, a spring or helical-like element extends by increasing its pitch [31] as it displaces each coil in order to axially extend. Because of this, we modeled three variations of the helical actuator (HA) (Fig. S5(a-c, a'-c')), each of them with a different coiling pitch, to determine the relationship between pitch increase and intraluminal deformation, as well as modeling the TA chamber (Fig. S5(d,d')). First, we developed 3D models of the soft actuators on Fusion 360 (Autodesk®) and then, imported them into Abaqus/CAE (Simulia, Dassault Systemes™). The actuators models were meshed using quadratic tetrahedral, 3D solid hybrid elements (C3D10H). To capture the hyperelastic behavior of silicone, we used the Ogden material model, which parameters are described in [32]. These settings were kept constant across all simulations using 3D Solid objects. Boundary conditions and loads are represented in Fig. S5(a'-d').

S3.1 Intraluminal Deformation of the Helical Actuator

By comparing the top (Fig. S6(a-c)) and front views (Fig. S6(a'-c')) of the helical chambers, it can be observed that the intraluminal area tends to deform in a drop-like shape and decrease when pressurized. This size reduction is confirmed by Fig. S6(e) and (f) that shows that the intraluminal area and circumference of the helical chambers decreases with pressurization. A helical chamber with a pitch of 9 mm increases its intraluminal area at a pressure of 16 kPa by 0.5%, however, it decreases by 12%, 37% and 66% at 18, 20 and 22 kPa respectively. When the pitch is 18 mm, the intraluminal area is reduced by 3%, 17%, 41%, and 70% respectively, and when the pitch is 36 mm, the intraluminal area is reduced by 5%, 20%, 43% and 69% respectively. Since the changes in area might not suffice to describe the deformation of the intraluminal area, we also measured the changes

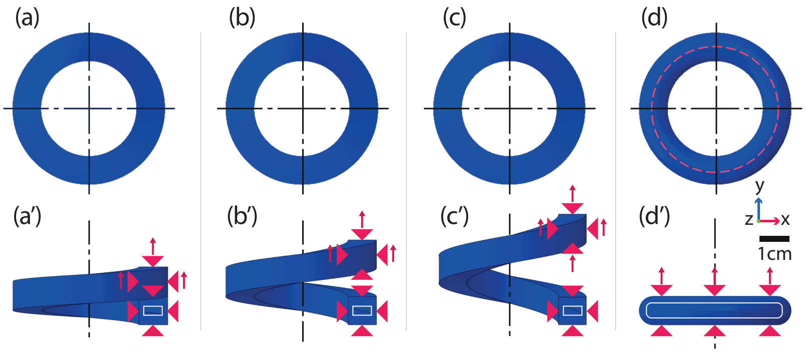

Figure S5: Numerical modeling setup for the evaluation of intraluminal deformation showing geometries and boundary conditions. (a-d) Top and (a'-d') front view of the relaxed helices with a pitch of 9, 18 and 36 mm and a torus respectively. For clarity, the boundary conditions of the torus were also marked as a dashed line in the top view. The load was applied throughout the channels in the chambers, which cross-sectional profile is highlighted as a solid line.

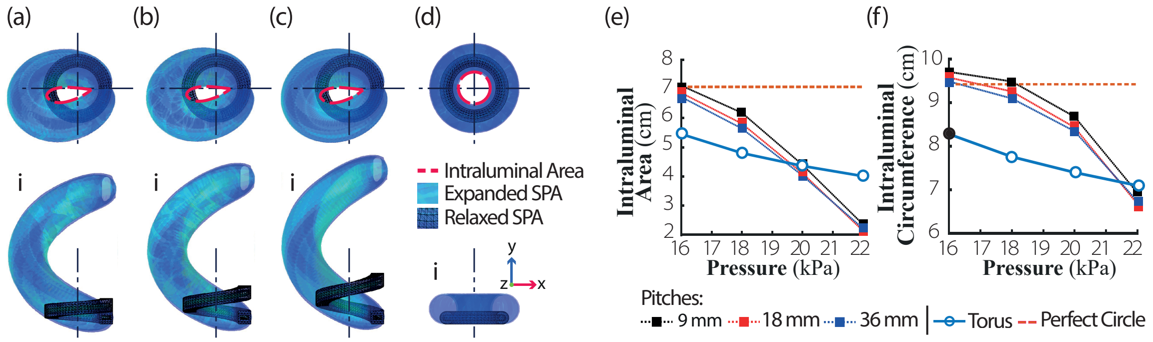

Figure S6: Intraluminal deformation of unconstrained helices and a torus. (a-c) Top and (a'-c') front views of the actuated helices with a pitch of 9, 18 and 36 mm respectively at 22 kPa of pressure. (d) Top and (d') front view of an actuated torus also actuated at 22 kPa of pressure. (e) Intraluminal deformation in function of pressure based on area and (f) circumference. The dashed lines in both (e) and (f) show the ideal behavior of the actuators for the clinical application, in which the luminal area remains undeformed regardless of the pressure conditions which is the ideal scenario.

in circumference. A helical chamber with a pitch of 9 mm increases its intraluminal circumference by 3% and 0.67% at a pressure of 16 and 18 kPa respectively. However, it decreases by 7.9% and 26.4% at 20 and 22 kPa respectively. When the pitch is 18 mm, the helical intraluminal circumference increases by 1.6% at a pressure of 16 kPa. However, it decreases by 1.76%, 10.5% and 29% at 18, 20 and 22 kPa respectively. When the pitch is 36 mm, the intraluminal circumference increases by 0.67% at a pressure of 16 kPa. However, it decreases by 3.45%, 11.4% and 28.5% at 18, 20 and 22 kPa respectively.

S3.2 Intraluminal Deformation of the Toroidal Actuator

By comparing the top (Fig. S6(d)) and front views (Fig. S6(d')) of the toroidal chamber, it can be observed that the intraluminal area tends to keep its circular shape. However, Fig. S6(e) and (f)

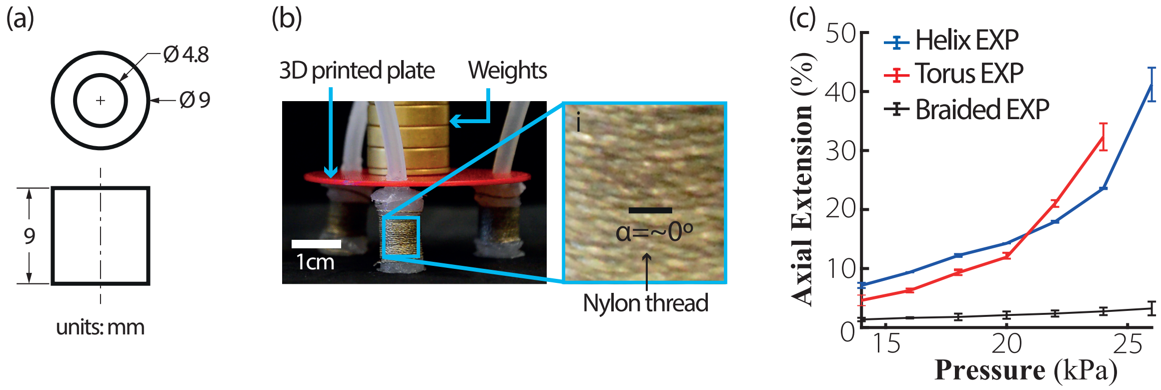

Figure S7: Braided actuator: (a) General dimensions and (b) experimental setup for the structural strength characterization. The axial extension characterization was conducted without the weights. (c) Plot showing a comparison of the axial extension performance under identical conditions between the M2H-HBMAs and the BAs.

show that there are variations in the intraluminal area and circumference in relation to the pressure applied to the chamber. The intraluminal area of the toroidal chamber decreases by 23%, 32%, 38% and 43% at 16, 18, 20 and 22 kPa respectively and its intraluminal circumference increases by 12%, 17%, 21% and 24% at 16, 18, 20 and 22 kPa respectively.

In summary, intraluminal deformation of the helical chambers is directly proportional to its inner pressure and the pitch between coils. However, the effects of the different pitch on the lumen deformation become similar at $\sim$ 22 kPa. The toroidal chamber showed to keep the circular shape consistently among pressures, with reductions in area and circumference of up to 43% and 24% respectively; 39% and 14% lower area and circumference reduction than the helical chamber. These results are an indication of the efficacy of the semi-soft exoskeletons used in the M2H-HBMAs to achieve pure-motions in the HA (Section 3.1.4).

S4 Comparison to braided pure-extension actuators

We fabricated a set of three pure-extension braided actuators (BA) with normalized dimensions (Fig. S7(a)) to one module of the M2H-HBMAs ((Fig. 3(b) and (Fig. 4(b)) to provide a preliminary comparison between to the actuators presented in this work in terms of extension and strength. Further details on the design and operating principle of the BAs can be found in [37]. The experimental setup is shown in Fig. S7(b). The results show that the BA can axially extend only 7.8% (Fig. S7(c)) and withstand up to 0.8 N of load before buckling under the same pressure and load conditions as the M2H-HBMAs. This represents 300% and more than a thousand percent lower axial extension and strength capabilities than the M2H-HBMAs respectively.

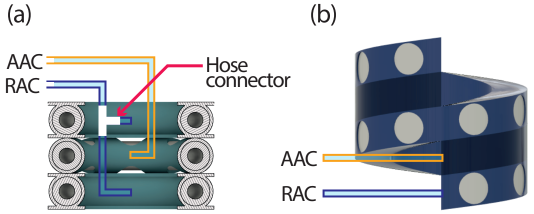

Figure S8: Diagrams showing the locations of the air inlets for the (a) TA and (b) HA.

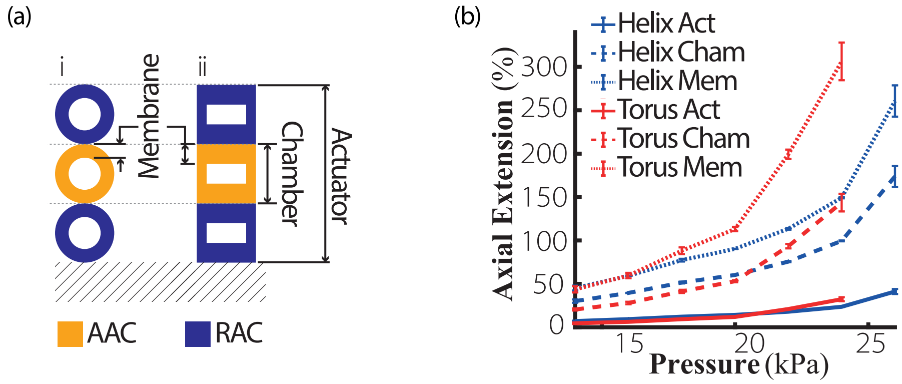

Figure S9: Axial extension capabilities of the M2H-HBMAs at actuator, chamber and membrane levels. (a) Schematic of the cross-sectional view of the M2H-HBMAs that identifies the experimentally measured sections highlight their extension capabilities. The exoskeletons were removed for clarity. (b) Plot comparing the extension capabilities of the membrane thickness, a chamber alone and as part of one level of the M2H-HBMAs.
